# Prevalence of Co-infection of Culture-Proven Bacterial Pathogens in Microbiologically Confirmed Pulmonary Tuberculosis Patients From a Tertiary Care Center

**DOI:** 10.7759/cureus.66482

**Published:** 2024-08-08

**Authors:** Raunak Bir, Rahul Ranjan, Jayanthi Gunasekaran, Kuhu Chatterjee, Dr Karteeka, Ankita Rai, Sonam Gupta, Priya Karlapudi, Ina Joshi, Rajiv M Gupta

**Affiliations:** 1 Department of Microbiology, ESIC (Employees' State Insurance Corporation) Medical College and Hospital, Faridabad, IND

**Keywords:** community acquired, indeterminate, sensitive, resistant, co-infection, sputum, mycobacterium tuberculosis complex, bacterial culture, cbnaat ultra, pulmonary tuberculosis

## Abstract

Tuberculosis (TB) is a chronic condition that weakens the immune system, causes structural changes in the lungs, and can lead to infections by other bacterial pathogens. Very few studies have been done to understand the magnitude of co-infection with other bacterial pathogens, so this study was conducted to understand the co-infection pattern and burden.

A total of 174 microbiologically confirmed pulmonary TB patients' samples, identified by cartridge-based nucleic acid amplification test, were further tested for other bacterial pathogens by culture over a period of five months from May 2023 to September 2023. The isolates' identification and drug susceptibility were performed using the VITEK 2 system (bioMérieux, Marcy-l'Étoile, France).

Of the 174 pulmonary samples tested, 19 samples grew a significant amount of other bacterial pathogens, making the prevalence 10.91% (19/174). Among the pulmonary samples tested, 54.59% were sputum, 38.5% were bronchoalveolar lavage, and 6.89% were endotracheal aspirate. Additionally, 70.11% of the patients tested were in the age group of 19-60 years. Of the patients who had co-infection, 94.73% (18/19) were male. The most common bacterial infection was caused by *Pseudomonas aeruginosa*, which was identified in 36.84% of the co-infection cases (7/19). This was followed by *Acinetobacter baumannii *in 31.57% (6/19), *Klebsiella pneumoniae *in 26.31% (5/19), and *Stenotrophomonas maltophilia *in 5.28% (1/19). *Acinetobacter baumannii *and *Klebsiella pneumoniae *showed high drug resistance, ranging from 60% to 100% against various groups of drugs tested. None of the patient samples with co-infection showed rifampicin resistance. Among all the samples with co-infection, the majority (42.10%, or 8/19) had a high load of *Mycobacterium tuberculosis* complex detected by CBNAAT Ultra (Cepheid, Sunnyvale, California).

*Pseudomonas aeruginosa*, *Acinetobacter baumannii*, and *Klebsiella pneumoniae *are unusual pathogens causing infection in community patients and are known to cause illness in hospitalized patients. These organisms' resistance was also similar to the resistance shown by hospital-acquired infections. This indicates that bacterial co-infection in pulmonary TB patients will be similar to the pattern of hospital-acquired infections. The high prevalence of bacterial co-infections (10.91%) in patients with pulmonary TB poses a significant challenge as these bacterial pathogens are not susceptible to anti-tubercular drugs. Therefore, comprehensive screening for other bacterial infections in all pulmonary TB patients is crucial for effective treatment and outcomes.

## Introduction

In 2022, approximately 10.6 million people worldwide were diagnosed with tuberculosis (TB), an increase from the estimated 10.3 million cases in 2021 and 10.0 million cases in 2020. Thirty high TB burden countries accounted for 87% of the world’s TB cases in 2022, and two-thirds of the global total was in eight countries: India (27%), Indonesia (10%), China (7.1%), the Philippines (7.0%), Pakistan (5.7%), Nigeria (4.5%), Bangladesh (3.6%), and the Democratic Republic of the Congo (3.0%) [1].

India accounts for over a quarter of the world’s new TB cases. In 2022, approximately 2.8 million people were newly affected by TB in India. Tragically, an estimated 342,000 deaths occurred due to TB during that year, equating to 39 citizens losing their lives to TB every hour [1].

India is a country with a high burden of TB, which is a chronic condition that weakens the immune system and makes patients susceptible to many other infections [2]. The co-occurrence of TB and bacterial infections in immunocompetent patients is rarely documented. When such a co-infection occurs, it exacerbates the patient’s clinical condition. Treating patients with co-infection becomes challenging due to the distinct treatment approaches required for each condition [3]. Given the scarcity of global studies on the co-occurrence of TB and bacterial infections, this research provides valuable insights. Notably, no such study has been conducted in India. By assessing the prevalence of bacterial pathogens in confirmed pulmonary TB cases, this study can improve the diagnostic strategies for suspected co-infection patients.

## Materials and methods

Aims and objectives

The aim of the study was to determine the prevalence of co-infection of bacterial pathogens in microbiologically confirmed pulmonary TB patients; to explore the correlation between rifampicin resistance and co-infection with bacterial pathogens; and to assess the susceptibility patterns of bacterial pathogens isolated from these patients.

Study population

This study was conducted in the Department of Microbiology, ESIC (Employees' State Insurance Corporation) Medical College and Hospital, Faridabad, India, from May 2023 to September 2023.

Inclusion criteria

All presumptive pulmonary TB patients' samples from all age groups that were tested for TB and microbiologically confirmed by cartridge-based nucleic acid amplification test (CBNAAT) - Xpert MTB/RIF Ultra (Cepheid, Sunnyvale, California) were included in the study.

Exclusion criteria

All the samples where *Mycobacterium tuberculosis *complex (MTBC) was not detected by CBNAAT - Xpert MTB/RIF Ultra were excluded from the study.

Sample size

A sample size calculation was not conducted, as this was a novel study with no existing data on the prevalence of co-infection of bacterial pathogens in microbiologically confirmed pulmonary TB patients in India. Therefore, a convenient sample of 174 pulmonary samples (sputum, tracheal aspirate, bronchoalveolar lavage) collected over a period of five months was included in the study, all of which were microbiologically confirmed for TB.

Sample processing

The samples were further processed for aerobic culture to detect other bacterial pathogens. Samples were cultured on blood agar and MacConkey agar at 37ºC overnight. Bacterial growth was considered pathogenic if significant numbers were detected, specifically ≥10^5^ CFU/mL for sputum and tracheal aspirate samples, and ≥10^3^ CFU/mL for bronchoalveolar lavage samples. These pathogenic bacterial isolates were then identified, and antimicrobial susceptibility testing was performed using the VITEK 2 system (bioMérieux, Marcy-l'Étoile, France).

Data collection

Data were recorded from patient requisition forms, which included demographic details such as name, age, sex, residential address, and date of registration, as well as laboratory details such as the date of sample collection, date of sample processing, date of reporting, type of sample, and test results.

The results of CBNAAT - Xpert MTB/RIF Ultra were recorded as MTBC detected or not detected, followed by rifampicin resistance detected or not detected, and the load of MTBC categorized as high, medium, low, very low, or trace.

Other pathogenic bacterial culture results were recorded with the identification of the pathogen and its susceptibility pattern to various drugs, including amoxicillin-clavulanic acid, ceftazidime, cefepime, cefixime, cefoperazone, piperacillin-tazobactam, levofloxacin, imipenem, meropenem, gentamicin, minocycline, and cotrimoxazole, classified as either sensitive or resistant.

Data analysis

All the collected data were recorded in MS Excel (Microsoft Corporation, Redmond, Washington), and the results were analyzed according to the study's objectives.

## Results

Of the 174 pulmonary samples microbiologically confirmed for MTBC by CBNAAT, 19 samples grew bacterial pathogens. The prevalence of bacterial coinfection in pulmonary TB patients was 19/174 (10.91%). Among the patient samples tested, 153/174 (87.93%) were male, and 21/174 (12.06%) were female (see Table 1).

**Table 1:** Gender distribution among the patients tested for co-infection of Mycobacterium tuberculosis complex and other bacterial pathogens (N = 174).

Gender	n (%)
Male	153 (87.93)
Female	21 (12.06)

Among the pulmonary samples tested, sputum was the most common sample type, accounting for 95/174 (54.59%), followed by bronchoalveolar lavage (BAL) at 67/174 (38.50%) and endotracheal aspirate at 12/174 (6.89%) (see Table 2).

**Table 2:** Distribution of aample types among the samples tested for co-infection of Mycobacterium tuberculosis complex and other bacterial pathogens (N = 174).

Sample Type	n (%)
Sputum	95 (54.59)
Broncho alveolar lavage (BAL)	67 (38.50)
Endotracheal aspirate	12 (6.89)

Among the samples tested, 122/174 (70.11%) were from patients in the age group of 19-60 years (see Table 3).

**Table 3:** Age group distribution of patients tested for co-infection of Mycobacterium tuberculosis complex and other bacterial pathogens (N = 174).

Age Group, Years	n (%)
<18	8 (4.59)
19–60	122 (70.11)
>60	44 (25.28)

Among the bacterial pathogens isolated, *Pseudomonas aeruginosa *was the most common, accounting for 7/19 (36.84%) samples, followed by *Acinetobacter baumannii *at 6/19 (31.57%), *Klebsiella pneumoniae *at 5/19 (26.31%), and *Stenotrophomonas maltophilia *at 1/19 (5.28%) (see Table 4). Among the samples in which *Pseudomonas aeruginosa* was isolated, 71.42% (5/7) were from sputum, followed by endotracheal aspirate and bronchoalveolar lavage at 14.28% (1/7) each. For *Acinetobacter baumannii*, 66.66% (4/6) were isolated from sputum, with endotracheal aspirate and bronchoalveolar lavage each accounting for 16.66% (1/6). In the case of *Klebsiella pneumoniae*, 80% (4/5) were isolated from sputum, while 20% (1/5) were from endotracheal aspirate. The *Stenotrophomonas maltophilia *isolate was 100% (1/1) from a sputum sample.

**Table 4:** Bacterial pathogens isolated in pulmonary tuberculosis patients (N = 19).

Bacteria Isolated	n (%)
Pseudomonas aeruginosa	7 (36.84)
Acinetobacter baumannii	6 (31.57)
Klebsiella pneumoniae	5 (26.31)
Stenotrophomonas maltophila	1 (5.28)

None of the patients with bacterial coinfection showed rifampicin resistance (0%). *Pseudomonas aeruginosa *isolates exhibited drug resistance ranging from 15% to 60% across all the drugs tested. *Klebsiella pneumoniae *isolates showed drug resistance ranging from 60% to 100%, and *Acinetobacter baumannii *isolates demonstrated drug resistance ranging from 66% to 100% (see Table 5).

**Table 5:** Drug susceptibility pattern of the bacterial isolates.

Drugs	*Pseudomonas aeruginosa* (N=7) Sensitive, n (%)	*Acinetobacter baumannii* (N=6) Sensitive, n (%)	*Klebsiella pneumoniae* (N=5) Sensitive, n (%)	*Stenotrophomonas maltophilia* (N=1) Sensitive, n (%)
Amoxycillin clavulanic acid	-	-	0 (0)	-
Ceftazidime	3 (42.85)	2 (33.33)	-	-
Cefepime	4 (57.14)	2 (33.33)	-	-
Piperacilin tazobactam	4 (57.14)	1 (16.66)	0 (0)	-
Levofloxacin	5 (71.42)	1 (16.66)	0 (0)	1 (100)
Imipenem	6 (85.71)	0 (0)	0 (0)	-
Meropenem	6 (85.71)	0 (0)	0 (0)	-
Gentamicin	-	1 (16.66)	2 (40)	-
Minocycline	-	4 (66.66)	-	1 (100)
Cefixime	-	-	0 (0)	-
Ceftriaxone	-	-	0 (0)	-
Cefoperazone	-	-	0 (0)	-
Cotrimoxazole	-	-	-	0 (0)

The load of MTBC detected by CBNAAT in the samples with coinfection varied from high to trace. Most of the samples showed a high MTBC load, detected in 8/19 (42.10%) of the cases, followed by a low load in 7/19 (36.84%) of the cases (see Table 6).

**Table 6:** Mycobacterium tuberculosis complex load by CBNAAT - Xpert MTB/RIF Ultra (N = 19). CBNAAT - Xpert MTB/RIF Ultra, in addition to detecting *Mycobacterium tuberculosis* complex and rifampicin resistance, also quantifies the mycobacterial load as High, Medium, Low, Very Low, and Trace.

Load	n (%)
High	8 (42.10)
Medium	1 (5.26)
Low	7 (36.84)
Very low	2 (10.52)
Trace	1 (5.26)

## Discussion

In clinical settings, mixed pulmonary infections, particularly involving bacterial and fungal pathogens, are commonly encountered, especially in the lower respiratory tract [4,5]. Diagnosing mixed pulmonary infections is challenging due to overlapping imaging features and the lack of specificity in clinical manifestations [5]. Moreover, the extended empirical use of broad-spectrum antibiotics has led to the emergence of drug resistance in specific pathogens, resulting in an anticipated increase in drug-resistant strains [6].

Microorganisms do not exist in isolation; they consistently engage in direct or indirect communication with one another [7]. Patients infected with multiple pathogens exhibit a distinct immune response compared to those with a single pathogen infection, potentially resulting in varying clinical outcomes. Currently, limited research has explored these interactions, particularly within the respiratory system [8].

TB is responsible for more deaths than any other single infectious agent. It is caused by the bacterium *Mycobacterium tuberculosis *[1]. TB is associated with an elevated risk of co-infection due to immune dysfunction. Structural changes in the lung parenchyma, including bronchiectasis, cicatrization, and scarring, can impact normal pulmonary function. When *Mycobacterium tuberculosis *co-infects with other bacterial pathogens, such as *Klebsiella *and *Pseudomonas*, it can exacerbate the severity of TB and elevate the risk of complications. Co-infections can result in more extensive lung damage and necessitate longer treatment durations [9]. The epidemiology and best diagnostic approaches for co-infection are still under investigation [6].

In regions with high TB prevalence, where TB treatment often relies on presumptive diagnosis, data on the co-infection of TB with other pathogens remain scarce [10,11]. Given the lack of studies on co-infection in India, a country with a high burden of TB, this research could provide valuable insights into the actual prevalence of bacterial co-infections among known pulmonary TB cases. Such information may guide testing and treatment strategies for suspected co-infection patients. The prevalence of bacterial co-infection in pulmonary TB patients in our study was 10.91%. In another study by Attia et al., 33% of TB patients were found to have bacterial co-infection [9]. Similarly, Liu et al. observed that 31.4% of patients had both TB and bacterial co-infection [12]. In a study conducted by Kim and colleagues, 19% of patients with endobronchial TB had bacterial co-infections [13]. Ishikawa et al. reported that microorganisms other than normal oral flora were concurrently isolated from sputum in 16.8% of TB patients [14]. The higher prevalence reported in the studies mentioned above may be attributed to the use of molecular methods for detecting bacterial co-infections, whereas our study relied solely on culture to identify co-infection pathogens.

Among the patient samples tested in our study, 153/174 (87.93%) were male, and 21/174 (12.06%) were female. Additionally, 122/174 (70.11%) of the samples were from patients in the age group of 19-60 years. In Liu et al.'s study, 81.6% of the patients were male, and the median age was 56.8 years, findings that are similar to our study [12]. This similarity may be due to the higher exposure of males and the productive age group to infected environments.

Among the bacterial pathogens isolated in our study, *Pseudomonas aeruginosa *was the most common, accounting for 7/19 (36.84%), followed by *Acinetobacter baumannii *6/19 (31.57%), *Klebsiella pneumoniae *5/19 (26.31%), and *Stenotrophomonas maltophilia *1/19 (5.28%). Attia et al. reported similar findings, where *Klebsiella pneumoniae *and *Pseudomonas aeruginosa *were the predominant causes of respiratory infections, both in cases with and without TB co-infection [9]. In Ishikawa et al.'s study, the most prevalent microorganisms included methicillin-resistant *Staphylococcus aureus*, *Klebsiella pneumoniae*, *Pseudomonas aeruginosa*, and methicillin-sensitive *Staphylococcus aureus*, among others [14]. Liu et al. confirmed that 28% of the patients had co-infections involving both *Klebsiella pneumoniae *and *Mycobacterium tuberculosis *[12]. In the study by Kim and colleagues, *Staphylococcus aureus* was the most prevalent respiratory microorganism in patients with endobronchial TB, accounting for 33.3% of cases with detected microorganisms, followed by *Klebsiella pneumoniae* and *Streptococcus species *[13]. In our study, we did not isolate *Staphylococcus* and *Streptococcus *species, which is likely due to repeated hospital visits where patients may have acquired gram-negative pathogens. The higher relative abundance of gram-negative bacteria cultured from respiratory specimens may be attributed to antibiotic pre-treatment within our population. The presence of gram-negative bacteria cultured from sputum could also indicate colonization of abnormal lung architecture resulting from TB, which later became pathogenic and caused infections when the patients' immunity was lowered due to TB.

Physicians need to recognize that pneumonia caused by organisms like *Pseudomonas aeruginosa*, *Acinetobacter baumannii*, *Klebsiella pneumoniae*, and *Stenotrophomonas maltophilia *can occur as a co-infection in patients with pulmonary TB (PTB) [12]. Distinguishing these co-infections is crucial for precise TB diagnosis, selecting appropriate treatment, and implementing effective control measures. Delayed diagnosis of co-infections may contribute to patient deterioration and necessitate multiple treatment modifications.

In our study, all *Acinetobacter baumannii *(6/6, 100%) and *Klebsiella pneumoniae *(7/7, 100%) isolates exhibited carbapenem resistance. However, among *Pseudomonas aeruginosa *isolates, only 1 out of 7 (14.3%) showed carbapenem resistance. In Liu et al.'s study, 12.5% of *Klebsiella pneumoniae *strains isolated from patients demonstrated carbapenem resistance. Previous research has confirmed that infections caused by carbapenem-resistant microbes are associated with a higher mortality rate [12,15].

A delayed diagnosis of co-infection can lead to prolonged inflammatory responses in patients [6]. According to Ishikawa et al., individuals with pulmonary TB who are co-infected with other microorganisms tend to have worse treatment outcomes and higher mortality rates compared to patients with TB alone. Specifically, the mortality rates were 39.8% (51 out of 128) for co-infected patients and 10.2% (59 out of 580) for those without additional microorganisms [14]. In a study conducted by Liu et al. [12], the number of patients who failed treatment was significantly lower (only three patients) compared to the study by Ishikawa et al. [14]. This discrepancy could be attributed to Liu et al.’s focus on only TB and Klebsiella pneumoniae co-infection. Notably, all three patients who failed treatment in Liu J’s study also experienced respiratory failure, suggesting that respiratory failure may serve as a risk factor for unfavorable treatment outcomes in TB and *Klebsiella pneumoniae* co-infection.

Our study has certain limitations. Further clinical history and treatment follow-up are necessary to understand the clinical significance of the isolated bacterial pathogens and their outcomes. Additionally, we were solely dependent on culture for detecting the pathogens, which precluded the determination of information related to non-culturable bacteria and viruses. We did not exclude patients who had received antibiotics before the study, raising concerns that culture results may have yielded false negatives in such cases. As our study was conducted in a laboratory setting, we did not evaluate treatment follow-up and outcomes of the patients.

The advantage of our study was that it was planned to isolate the bacterial pathogens by culture rather than by molecular methods, as molecular methods can detect even colonizers, which may not be of clinical significance. In our study, only cultures that grew pathogens in significant numbers were considered relevant.

To gain comprehensive insights into the occurrence and prevalence of pulmonary TB with co-infections involving other pathogens, additional investigations with larger sample sizes and data from multiple centers are necessary.

## Conclusions

Our study investigated the culture-positive bacterial co-infection among pulmonary TB patients, revealing a high prevalence of such co-infections. The prevalence of bacterial co-infection in pulmonary TB patients was 10.91%, with *Pseudomonas aeruginosa *emerging as the most common pathogen, followed by *Acinetobacter baumannii*. By evaluating the prevalence of bacterial pathogens in confirmed pulmonary TB cases, this research contributes to refining diagnostic and treatment strategies for suspected co-infection patients. A significant proportion of the bacterial strains isolated in our study exhibited multidrug resistance, highlighting the critical need for focused attention on managing multidrug-resistant infections. These cases often present with heightened inflammatory responses, emphasizing the importance of providing adequate respiratory support, timely clinical intervention, and antibacterial treatment tailored to the susceptibility pattern. Customizing treatment regimens based on individual patient profiles becomes crucial for achieving favorable outcomes in patients with co-infection TB cases.
